# Social Isolation and Loneliness: Moderators of the Relationship Between Sensory Impairment and Cognition

**DOI:** 10.1093/geroni/igaa057.1534

**Published:** 2020-12-16

**Authors:** Alison Huang, George Rebok, Bonnielin Swenor, Jayant Pinto, Linda Waite, Jennifer Deal

**Affiliations:** 1 Johns Hopkins Bloomberg School of Public Health, Baltimore, Maryland, United States; 2 Johns Hopkins University, Baltimore, Maryland, United States; 3 University of Chicago, Chicago, Illinois, United States

## Abstract

Hearing and vision impairment have been independently linked to accelerated cognitive decline in older adults, however there is limited evidence on the effect of dual sensory impairment (DSI) (both hearing and vision impairment) on cognition. Additionally, the impact of social isolation and loneliness, both correlates of DSI and independent risk factors for cognitive decline, on the DSI-cognition relationship has yet to be studied. Using data from the National Social Life, Health, and Aging Project (N=3,091), multivariable linear regression models were used to describe the cross-sectional relationship between self-reported functional sensory impairment (none, hearing only, vision only, DSI) and cognitive function, measured by the survey adapted Montreal Cognitive Assessment. We also included an interaction term in the model to investigate whether cognition is worse among older adults with sensory impairment who also are socially isolated or lonely. Participants in this sample are between 62-91 years with 15% reporting hearing impairment, 11% reporting vision impairment, and 7% reporting DSI. DSI was associated with significantly lower global cognitive function compared to no sensory impairment (-0.31 standard deviations (SD), 95% CI:-0.44 to-0.18), hearing impairment alone (-0.29 SD, 95% CI: -0.44 to -0.15), and vision impairment alone (-0.22 SD, 95% CI: -0.39 to -0.06). Furthermore, cognitive function was significantly worse among older adults with both DSI and smaller social networks (p-interaction <0.05). No differences in the DSI-cognition relationship were observed by level of loneliness. These findings add to the limited research on the relationship between DSI, social isolation and loneliness, and cognition.

